# Thermal care for newborn babies in rural southern Tanzania: a mixed-method study of barriers, facilitators and potential for behaviour change

**DOI:** 10.1186/1471-2393-14-267

**Published:** 2014-08-11

**Authors:** Donat Shamba, Joanna Schellenberg, Zoe Jane-Lara Hildon, Irene Mashasi, Suzanne Penfold, Marcel Tanner, Tanya Marchant, Zelee Hill

**Affiliations:** Ifakara Health Institute, P.O. Box 78373, Dar Es Salaam, Tanzania; Faculty of Infectious and Tropical Diseases, London School of Hygiene and Tropical Medicine, London, UK; Center for International Health and Development, Institute of Child Health, University College London, London, UK; Swiss Tropical and Public Health Institute, Basel, Switzerland; Saw Swee Hock School of Public Health, National University Singapore, Singapore, Singapore; Faculty of Public Health & Policy, London School of Hygiene and Tropical Medicine, London, UK

**Keywords:** Thermal care, Hypothermia, Newborn, Formative research, Behaviour change, Mixed methods

## Abstract

**Background:**

Hypothermia contributes to neonatal morbidity and mortality in low-income countries, yet little is known about thermal care practices in rural African settings. We assessed adoption and community acceptability of recommended thermal care practices in rural Tanzania.

**Methods:**

A multi-method qualitative study, enhanced with survey data. For the qualitative component we triangulated birth narrative interviews with focus group discussions with mothers and traditional birth attendants. Results were then contrasted to related quantitative data. Qualitative analyses sought to identify themes linked to a) immediately drying and wrapping of the baby; b) bathing practices, including delaying for at least 6 hours and using warm water; c) day to day care such as covering the baby’s head, covering the baby; and d) keeping the baby skin-to-skin. Quantitative data (n = 22,243 women) on the thermal care practices relayed by mothers who had delivered in the last year are reported accordingly.

**Results:**

42% of babies were dried and 27% wrapped within five minutes of birth mainly due to an awareness that this reduced cold. The main reason for delayed wrapping and drying was not attending to the baby until the placenta was delivered. 45% of babies born at a health facility and 19% born at home were bathed six or more hours after birth. The main reason for delayed bathing was health worker advice. The main reason for early bathing believed that the baby is dirty, particularly if the baby had an obvious vernix as this was believed to be sperm. On the other hand, keeping the baby warm and covered day-to-day was considered normal practice. Skin-to-skin care was not a normalised practice, and some respondents wondered if it might be harmful to fragile newborns.

**Conclusion:**

Most thermal care behaviours needed improving. Many sub-optimal practices had cultural and symbolic origins. Drying the baby on birth was least symbolically imbued, although resisted by prioritizing of the mothers. Both practical interventions, for instance, having more than one attendant to help both mother *and* baby, and culturally anchored sensitization are recommended.

## Background

Despite a reduction in neonatal mortality in recent years [1, 2] levels remain unacceptably high globally. Most deaths occur in lower-income countries and in home settings [3]. Community interventions of known efficacy exist [4, 5] and WHO recommends home visits for all newborns in the first week of life to promote recommended care behaviours [6]. These behaviours include several relating to the prevention of hypothermia, which is a major contributor to newborn morbidity even in tropical areas [7]. Low birth weight greatly increases the risk of hypothermia, and is most common in developing countries [5]. Recommended thermal care practices include immediate drying and wrapping, skin-to-skin contact after birth, delaying bathing until the second day of life, appropriate dressing of the baby and immediate and frequent breastfeeding [8–13].

Other existing studies on thermal practices in Ghana and Uganda report strong cultural beliefs, in these cases about the benefits of early bathing [14, 15], which determine uptake. Also, a fear of transmission of HIV/AIDS has been reported as a barrier to skin-to-skin care in high prevalence settings [16, 17]. Accordingly, formative research seeking to understand beliefs and practices that drive behaviour has been found to be essential for designing effective interventions [18]. The Improving Newborn Survival in Southern Tanzania (INSIST) project was conceived in 2006 as a study to develop, implement and evaluate a scalable behaviour-change intervention at community level to improve newborn care and reduce neonatal mortality in an area of Tanzania where neonatal mortality was higher than the national average [19]. In order to develop an appropriate counselling strategy, formative research was undertaken, including a household survey to determine the frequency of newborn care behaviours and qualitative research, to understand reasons for behaviours and the potential for change [20]. Here we report the findings from the qualitative research relating to thermal care practices alongside the frequency on these behaviours extracted from the household survey.

Our aim is to improve understanding of the motivations behind adopting - or not adopting - thermal care practices in the Tanzanian home and healthcare facility contexts. Findings will inform assessment of future uptake of such behaviours and behaviour change interventions. Our objectives were to:

1. Explore perception and experiences linked to four thermal care practices: a) immediately drying and wrapping of the baby; b) bathing practices, including delaying for at least 6 hours and using warm water; c) day to day care such as covering the baby’s head; and d) keeping the baby skin-to-skin.
2. Contrast these findings with available baseline quantitative data describing the extent to which they were reportedly adopted in informal or institutional settings.

## Methods

We undertook a mixed-method study using predominantly multiple qualitative methods, reported below following the Consolidated Criteria for Reporting Qualitative Research (COREQ) [21]. Qualitative themes are contrasted to household survey data (243,612) on thermal care, although the methods and main results of the survey are fully reported elsewhere [20]. The qualitative study took place 2008 (September to December) and was preceded by the quantitative survey carried out in 2007 (June and October). The survey included consenting female residents’ age 13–49 that had a live birth within the year preceding the survey. Qualitative methods included birth narrative interviews, in-depth interviews (IDIs) and focus group discussions (FGDs) with mothers and traditional birth attendants (TBAs - meaning any woman who assisted home birth in the last one year including trained and untrained traditional birth attendants). In Tanzania the last formal traditional birth attendants training were done in 1980s.

The multiple methods allowed for triangulation of results, but also allowed us to draw on the strengths of each [22]: the quantitative data giving us an idea of acceptability of practices, and the qualitative allowing us to explore the credibility and reasons behind these frequencies.

### Study area

The study was conducted in Lindi and Mtwara regions in Southern Tanzania, which is a largely rural area, comprised of plateaus, mountainous areas, low lying plains and coastal areas. The population is comprised of mixed ethnic groups who speak the language of their own ethnic group and Swahili. The temperatures are consistently hot and humid, averaging around 30 degrees Celsius, [23]. The most common occupations are subsistence farming, fishing and small-scale trading. Most people live with their extended families in mud walled and thatched-roof houses and get water from hand-dug wells, communal boreholes, natural springs or rivers. The health system has a network of government and NGO-owned facilities that reach to village level. At the time of the study most women attended antenatal clinic at least once in pregnancy and delivered at home, and no formal system existed for postnatal checks [20, 24, 25].

### Qualitative research team and reflexivity

Data were collected using semi-structured topic guides by one male and one female Tanzanian experienced graduate researcher (DS, and IM) trained in qualitative data collection. Where possible researcher and respondent characteristics were matched, for instance, the female researcher interviewed younger or shyer females; there was not prior relationship between the participants and researchers. Field assistant took notes during interviews and FGDs, some interviews and all FGDs were tape recorded for quality assurance purposes and to assist in writing up. DS and IM translated the tools (English into Swahili) and interview/FGD data (Swahili to English) and analysed the data. The interviewers themselves were chosen to carry out the translations to ensure that the original meanings conveyed by the participants were fully captured. The field team also undertook formal translation training on quality assurance, for example they were tasked with keeping a ‘vocabulary book’ to record local terminologies. A senior scientist and native English speaker (ZH) checked the English translations for clarity within 48 hours and final transcripts were agreed.

Analyses were undertaken on English *expanded notes* [26] captured in the third person, salient quotes were noted and translated verbatim in the first person.

### Qualitative study design

The theoretical underpinning of the study was inductive, applying principals of grounded theory [27, 28]. Using expanded notes allowed us to incorporate analysis into data collection as an iterative component to the study, and to use saturation sampling [29]. All interviews and FGDs were written up in full the same day into English in MS Word, using observational notes and available audio recordings. Topics that were covered included descriptions of thermal care practices, discussions around non-optimal practices and the acceptability of new practices, outlined in Table 1.

**Table 1:** **Summary of the qualitative methods**

Method	Description of the method	Content related to thermal care
**Birth narratives with mothers of babies below two months old (n = 11 for home births and 9 for facility deliveries)**	Women were asked to describe their recent delivery and newborn care practices.	(1) Preparations for birth (e.g. cloths for drying and wrapping the baby).
		(2) The birthing process including what happens to the baby immediately after delivery, timing and method of bathing and how the baby was dressed or covered.
**FGDs with mothers of children under two years of age (n = 6)**	Women were asked to share their experiences and views on newborn care behaviors and barriers and facilitators to change.	(1) whether and how problem behaviors can be changed
		(2) What compromise behaviors could be advocated.
**FGDs with TBAs – woman who had assisted a home birth in last year (n = 2), other 2 additional IDIs with TBAs were conducted with different respondents**	Female attendants were asked about the behaviors of all involved during delivery and the neonatal period.	(1) Home birthing process and what influences hygiene care practices.
		(2) The ‘gate keepers’ perspective on changing birthing behaviors and introducing ‘compromise’ behaviors.

Sampling was purposive, recruiting women who had either given birth or helped to give birth [29]. Recruitment was through key informants who lived in the study area (village leader or village health workers), for this reason non-participation was not recorded. Recruiters were, however, asked to invite people with a spread of socio-demographic characteristics, such age and income. Participants were recruited from three in villages in Lindi district selected to reflect diversity in ethnicity and access to health facilities.

Twenty birth narratives with mothers who had delivered in the last two months, six FGDs with mothers with children under two years, two FGDs with TBAs, and two complimentary in-depth interviews with TBAs were conducted. Sample characteristics are described in Table 2. All interviews were conducted in the home, in a private setting, and lasted between thirty minutes and sixty minutes. FGDs were conducted in empty classrooms, which were deemed a neutral setting, encouraging participants to speak freely. These sessions lasted up to one and a half hours.

**Table 2:** **Summary of sample characteristics**

	Number of participants			
	Mothers		TBAs	
	Birth narratives (n = 20)	FGDs (n = 37)	FGDs (n = 12)	In-depth interviews (n = 2)
**Age**				
15-24	9	10	0	0
25-34	7	13	1	0
35-44	4	13	2	0
45-54	0	1	2	2
55-64	0	0	3	0
65-74	0	0	3	0
75-84	0	0	1	0
**Ethnicity**				
Makonde	8	17	6	1
Mwera	4	15	5	1
Ndonde	4	1	1	0
Yao	2	3	0	0
Makua	1	0	0	0
Mnali	1	0	0	0
Ngindo	0	1	0	0
**Income of household**				
Very low	1			
Low	13	N/A	N/A	N/A
Moderate	6			

### Qualitative analysis and findings

After all data collection was carried out the complete data store, pooling narrative, IDIs and FGDs note form transcripts were shared and explored with senior members of the research team (ZH, SP, and JS). NVIVO software [30] was used to manage the data and facilitate thematic inter-rater coding. The researchers analyzed independently, coding first and second order concepts, and then discussed and agreed broad themes. Subsequently, this analysis were refined (by DS and ZJH) who anchored it to the first objective in this paper, organizing the data according to the popularity or acceptability of each behavior of interest, and cataloguing the implicit or explicit reasons given for this. Participant checking was not used, however minority themes and inconsistencies across data sources were discussed. As such, qualitative data collected using different methods and participant groups were triangulated throughout this process.

### Incorporated quantitative analyses

Mothers in all five districts of Lindi and Mtwara regions were asked questions relating to thermal care and other aspects of essential newborn care for the most recent birth (n = 22,243). Survey findings are reported elsewhere [20]; for the purpose of the current paper we re-analysed the thermal care data disaggregated into (a) deliveries taking place in a facility (meaning hospital, health centre or dispensary) and (b) at home (meaning the woman’s own home, and excluding deliveries in other households).

This paper reports data on all recommended thermal care practices except the initiation of breastfeeding as feeding behaviours differ substantially from drying, wrapping and bathing behaviours.

### Ethical approval

The study was part of the INSIST trial (http://www.clinicaltrials.gov, NCT01022788). INSIST, including the formative research, and received ethical approval from institutional review boards of Ifakara Health Institute, the National Tanzania Medical Research Co-coordinating Committee, the Tanzania Commission for Science and Technology, and the London School of Hygiene and Tropical Medicine, UK. We obtained oral and written consent at the start of each interview and FGD and assured the anonymity and confidentiality of participants.

## Results

A total of 57 recently delivered women and 14 traditional birth attendants were included in the qualitative study; 58% of the recently delivered women had only primary education and 37% had never been to school; otherwise respondents ranged in age and ethnicity (Table 2).

We present the results according to four behaviours of interest: (a) drying and wrapping, (b) bathing practices, (c) day to day thermal care, and (d) skin-to-skin care. Quantitative data from the household survey are available for the first two behaviours, and are presented alongside the themes related to perceptions and experiences of using them, both in the home and healthcare facility settings.

### Drying and wrapping

From the household survey we found that 41% [3,731/9,043] of babies born in a facility were dried and 28% [2,548/9,043] were wrapped within 5 minutes of the birth with 12% of babies wrapped more than 15 minutes after delivery - figures were similar for home births. In the qualitative data ‘delay’ was judged by description of sequence of events, so it was hard to determine the exact time of delay, more how likely this would have been to exceed 5 minutes. And this was the case only in narratives that described home deliveries.

All women who delivered in facility reported that the baby was dried and wrapped straight away. Perhaps this was due to the effects of the programme and informal training promoting essential newborn care practices in the study areas. Relatedly, the TBAs could have felt compelled to appear agreeable to this practice during the interviews. Neverthless, this behaviour also seemed the least symbolically imbued compared to some of the other practices, which were linked instead to stigmatising beliefs, witchcraft or manhandling of the babies, so may be easier to change. Correspondingly, the main reason given for early wrapping and drying was an awareness that babies need protection from cold.

From the narratives most babies born at home were dried after the cord was cut and after the placenta was delivered. Waiting for the placenta was the main reason for delayed wrapping and drying, and was linked to a belief that the woman’s life is in danger until the placenta is delivered:

*‘After the baby was delivered, it took a half an hour until the house [placenta] was delivered. The baby was just laid between her legs’* [Narrative mother 25 year old, 2 children, educated to standard 7].

*‘Just after delivery the baby was put on a cloth between her mother’s legs to wait for the placenta to come out…nobody concentrated on the baby until the placenta came out’* [Narrative mother 41 year old, 4 children, educated to standard 7].

Less common reasons given from the narratives for delayed drying and wrapping were waiting for the baby to be bathed or an impromptu delivery. Focus group participants reported that if the baby did not cry after delivery drying and wrapping would also be delayed: *‘people here do not cover the baby until the baby gets fresh air and cries …… then they wrap the baby’* [FGD mother 18 year old, 1 child, educated to standard 7]; and also that having only one person present at the delivery makes attending to the baby early difficult as the mother needs attention.

Respondents felt behaviour change is possible with sensitization and education of those who assist in deliveries: *‘If people will be well educated they will wrap the baby even before the placenta comes out’* [FGD mother 40 year old, 5 children, educated to standard 7]. Potential barriers to behaviour change were a strong belief that the baby cannot be dried before the placenta is delivered, that delivery is complex and new behaviours may be hard to remember, and a resistance to change amongst older delivery assistants.

‘*To us deliver means when the placenta comes out, and we cut baby’s cord after the placenta comes out. We are worried whether people will accept that’* [FGD mother 44 year old, 2 children, educated to standard 7].

*‘Old women and women with birth experience are the ones who assist mothers to deliver, we are not sure if these two groups will be ready to change’* [FGD mother 45 year old, 2 children, educated to standard 7].

*‘If it happen there is only one person who is assisting … it will be difficult to remember to wrap the baby since she will be concentrating on helping the mother’* [FGD mother 44 year old, 2 children, educated to standard 7].

Practically speaking, the biggest barrier to drying and wrapping the baby quickly after delivery appeared to be not having enough birth attendants to help deal with both baby *and* mother at the same time. In general we observed certain fatalism about the survival of babies and infants, and a prioritising of the mother during the birthing process. Perhaps facility births were more likely to report drying and wrapping the baby compared to home birth because in the facility setting more people were available to attend to both parties needs.

### Bathing practices

In the household survey, 45% [4,060/9,043] of those delivering in a facility and 19% of those delivering at home reported waiting at least six hours before bathing. 43% (5,471/12,615) of babies bathed within six hours were bathed in cold water. They reported water temperature was variable.

From the qualitative data the main reasons for delayed bathing (5/8 narrative women) was health worker advice, with health workers reported as a key source of information with the recommended delay ranging from 5 hours to 3 days.

*‘The nurse told me not to bath the baby for two days to protect the baby from cold’* [Narrative mother 38 year old, 5 children, educated to standard 7].

*‘Pregnant women are told by the nurses at the dispensary to delay bathing the baby for the first day to protect it from cold’* [FGD TBA 49 year old, educated to standard 7];

*‘It is not like in the past, nowadays we are told at the dispensary not to bath the baby for three days after the baby’s birth’* [FGD mother 31 year old, 3 children, no schooling].

Other reasons for delayed bathing were waiting to return from the health facility and a belief that a newborn cannot be bathed at night or in cold weather: *‘If it is cold outside you can’t bath the baby because she will get cold’* [Narrative mother 30 year old, 4 children, educated to standard 7].

The main reason for early bathing was a belief that the birth process is dirty and that the baby is dirty after birth: *‘Babies are born with dirt on them; they need to be bathed’* [FGD mother 18 year old, 1 child, educated to standard 7]. The need to bath the baby quickly was particularly strong if there was an obvious vernix, this issue was raised spontaneously in the interviews with mothers. Probing the issue in FGDs confirmed the finding, and elucidated it as follows: the vernix was thought to be sperm *‘If the baby is born with some white things on their body, it shows that the mother was having sex when she was in the final months of her pregnancy, so the baby has to be washed to clean of that dirty stuff’* [FGD mother 30 year old, 1 child, educated to standard 7]. The desire to remove the dirt or vernix from the baby was related to social pressure for the baby to look smart or the mother not to appear sexualised, rather than a belief that the dirt can harm the baby:

*‘It is shameful for a mother to stay with the baby without bathing him or her’* [FGD mother 44 year old, 5 children, educated to standard 7].

*‘The belief that if the baby is born with white things on the skin means the baby is dirty with sperm, it means that women will not let the baby remain with those white things on skin because they will be feeling shy when people will come to see the baby’* [FGD mother 34 year old, 3 children, no schooling].

Narrative mothers were unsure why cold or warm water was used, while in the FGDs mother and TBAs reported that cold water helps the baby cry or makes them strong and warm water protects the baby from cold. Drying babies after bathing was seen as important, and was reported as universally practised.

Once initiated, baths were frequent due to social pressure: *‘They bath the baby two – three times a day, for fearing older mothers pointing at the mother as a dirty mother’* [FGD mother 30 year old, 1 child, educated to standard 7], or to keep the baby cool in hot weather *‘When the baby cries they say it is because of the hot weather and they decide to bath the baby’* [FGD mother 19 year old, 1 child, educated to standard 7].

Most respondents felt behaviour change was possible with sensitization *‘If people will be well educated they will follow [the advice]*’ [FGD mother 35 year old, 2 children, no schooling] or had already occurred due to health workers advice. The exception was in cases when vernix was visible – since its cultural meaning could lead to stigmatization of the mother and we found examples of families ignoring health workers advice when a vernix was visible:

*‘They took the baby outside the building secretly and bathed the baby. The nurse came suddenly and asked why they were bathing the baby when they were told not to. They said that they wanted to go home but couldn’t without bathing the baby because the baby was dirty with something from the womb, people in the village could say the baby was dirty. The baby had something white on its skin’* [Narrative mother 38 year old, 5 children, educated to standard 7].

Bathing the baby was usually undertaken by relatives such as aunts, sisters and grandmothers, who were considered important to target to change this behaviour: *‘Normally birth attendants do not bath babies, the mother of the new mother, her mother in-law or her aunt are the ones who bath the baby’* [FGD TBA 47 year old, no schooling].

### Day to day thermal care practices

Respondents in the FGDs and narratives reported that wrapping the baby well and keeping the head covered in the first week of life was important for keeping the baby warm, particularly if the weather was cold or the baby was being taken outside the house. *'She wrapped the baby with lots of cloth and also added a heavy towel to protect the baby from getting cold; she used other cloth to cover the head of the child'* [Narrative mother 25 year old, 2 children, educated to standard 7]. Mostly, newborns were said to be have been kept inside for at least the first week of life.

Keeping the head covered in the first week of life was also reported as protecting the baby from harmful intentions*: ‘the baby’s head was covered with a hat and he was clothed to protect his first hair from being seen by bad people so when the cord fell, they shaved his hair’* [Narrative mother 21 year, 1 child, educated to standard 7]. This experience was based on the belief that if the baby is not well covered in the first months of life, some people may be able to hurt it through witchcraft.

Respondents felt that behaviour change was not needed, as the behaviour was well understood and practiced.

### Keeping the baby skin-to-skin

Quantitative data on these practices were not collected as at the time of the data collection skin-to-skin practices were thought to be rare. We found no evidence of skin-to-skin care being practised during qualitative data collection.

During qualitative data collection mothers were asked about skin-to-skin care in the context of having a low birth weight infant and were shown a picture of a mother practicing skin-to-skin care. The concept of skin-to-skin puzzled women at first *‘This is new to us, we do not do that here’* [FGD mother 43 year old, 6 children, educated to standard 7]. Respondents understood the benefits of skin-to-skin care in terms of keeping the baby warm when these were explained and understood that the mothers warmth could help keep the baby warm: *‘It shows the mother can help the baby to be warm and babies need to be protected from cold* [FGD mother 28 year old, 2 children, educated to standard 6].

The main barriers given for skin-to-skin care, emerging in all FGDs with women, were concerns that keeping the baby skin-to-skin would injure the babies’ bones, give them chest problems or would hurt the cord:

*‘Not all people will like to put their babies on the chest because they think that the baby’s bones are not yet strong enough’* [FGD mother 34 year old, 3 children, no schooling].

*‘When the baby is born before the cord falls off, they don’t encourage people to carry the baby to avoid to injury’* [FGD mother 28 year old, 2 children, educated to standard 6].

Less frequently mentioned barriers were that women with birth problems would not be able to practice skin-to-skin *‘some women after birth they get stomach problem so it will be hard to put the baby on the chest’* [FGD mother 41 year old, 5 children, educated to standard 7]; that some after birth activities such as bathing and resting would delay initiation; that woman would be too busy with other tasks: *‘They will need to do other things like washing baby’s clothes and cooking for the* husband’ [FGD TBA 58 year old, no schooling]; that people may think there are other ways to keep the baby warm *‘Others will not follow; they might say that, the baby can be kept warm by wrapping with many cloths’* [FGD mother 34 year old, 3 children no schooling] and because the practice is not promoted at health facilities.

Views on the acceptability of behaviour change varied with some respondents saying that women will understand the importance of the practice and adopt it with education, detailed information and reassurance that it will not harm the baby: *‘We will follow but we need to educated on ….how to help the mother to put a baby on her chest’* [FGD TBA 48 year old, educated to standard 7]; but others felt that uptake would be slow: *‘It is a new thing people will not like to do it until they hear from other people who are doing and they could follow’* [FGD TBA 78 year old, no schooling].

## Discussion

Respondents often knew the importance of keeping babies warm and this was the main reason for positive thermal care behaviours [31, 32]. For those recommended thermal care behaviours not commonly practised, strong cultural beliefs were the main reasons given. For instance, we found that the need for the placenta to be delivered before the baby is attended to was a major factor in delaying drying and wrapping the newborn, although in itself attending to baby in this way was not objected to. We also found a strong belief that the baby is seen as dirty after delivery, particularly if the vernix was visible which led to stigmatising of the mother. There also seemed to be a fundamental belief that skin-to-skin care could harm the babies’ bones or cord. Conversely, we found that people wanted to cover the baby’s head; otherwise they believed that the newborn could be vulnerable to witchcraft.

Evidence of similar beliefs affecting thermal care of newborns has been found in other contexts. For example, delaying drying and wrapping the baby until the placenta was delivered has been reported in Nepal, Bangladesh, Pakistan and India [18, 33–37]. The need to make the baby clean and presentable through early bathing has been reported in Ghana and India [15, 37–40]. On the other hand, the belief that it was important to keep the baby well covered and not to bath with cold water was similarly found in Asia and Africa [15, 33–38, 41].

However, there were different perceptions relating to the potential to improve thermal care practices. In Uganda [14] mothers reported that bathing behaviours would be difficult to change due to the beliefs that babies are born dirty and with blood, babies who are not bathed smell bad and mothers prefer that visitors finds babies clean, however, evidence from Uttar Pradesh in India suggest that cultural beliefs can be overcome and behaviours can be changed, although the change may be gradual [4, 40, 42]. The stigma of a baby with a visible vernix has not been reported elsewhere and may be a particularly difficult barrier to overcome.

A recent large scale trial in Ghana found that home visits can change thermal care behaviours but gains were modest [43]. In the intervention zones, 44% practiced any skin-to-skin care compared to 24% in the comparison zones, and 41% delayed bathing for more than 6 hours in the intervention zones and 29% in the comparison zones [43]. One possible reason for the modest change was the conducting of the evaluation after a short implementation period of 14 months. Our study also suggests that there may be some resistance to skin-to-skin care and that adoption of this new behaviour may be gradual, with mothers needing to see others performing this behaviour. The length of time it will take for such a new behaviour to be adopted should be considered by those designing interventions and programmes.

Day to day thermal care was already being conducted adequately and should not be targeted in newborn care interventions in the area. Delayed bathing was strongly influenced by health workers but messages were not consistent. Efforts should be made to make messages uniform and to reach families who do not deliver in facilities. The need for consistent messages was also emphasized for skin-to-skin care as the fact that it is not done in facilities reported as a potential barrier to adoption of the behaviour.

Although mothers are key to thermal care interventions we also found that traditional birth attendants and family members are important to consider when designing an intervention to improve thermal care.

At times we observed a sense of fatalism about the survival of babies and infants, and a prioritizing of the mother during the birthing process. It is likely that the facility births reported drying and wrapping the baby more readily compared to home birth because in the facility setting there were more people available to attend to both the mother and the babies’ needs.

### Strengths and limitations

We note that despite the fact that both qualitative and quantitative studies were conducted in 2008 and 2007 respectively, problems affecting maternal and newborn babies still exist in the region [44]. In addition, our study remains relevant to formative study in comparable settings.

As for our methods, the combination of the quantitative and qualitative data meant that we understood both the frequency of behaviours and the reasons behind them. The fieldwork utilized well-trained researchers who were familiar with the setting and attuned to the culture, which may have helped put participants at ease and in building rapport. No obvious differences were found in the quality or depth of data from the male and female researcher suggesting that their gender did not influence responses. Data quality was assured by daily reviewing of interview transcripts between the research team, and translation being conducted by the field researchers shortly after collection.

The findings are limited to the specified set of practices, we did not present breastfeeding data. Our findings are also limited to Tanzanian rural settings and may vary in other ethnic groups, and by climate. For instance northern Tanzania is much cooler in the winters and may lead to different practices. Nevertheless, we have captured valuable insights into how thermal care for neonates might be encouraged in the south. Although each of the data collections methods we used has their weaknesses - notably the self-reporting of things like water temperature, or timing between drying in the survey - comparing and contrasting data from different methods helped to better unpack their meaning, and enhanced interpretability and hence the credibility of our analyses. For instance, we argue that delayed bathing was likely to have been over reported in the survey, as some women qualitatively noted that the babies were sometimes bathed in secret.

### Conclusion

Overall, reasons for sub-optimal thermal care practices were anchored in traditional or culturally held beliefs, but also at times in a lack of support – particularly during the birthing event. Therefore, both practical interventions, for instance recommendations to have more than one birth attendant, perhaps helped by a female relative, during deliveries, as well as culturally anchored sensitization storyboards are recommended.

Nevertheless, we found that some issues like prioritizing the mother or waiting for the baby to cry before wrapping it were described as ‘just done’ , and the reasons why were not spontaneously articulated by the women we spoke to. Future studies may seek to probe such behaviours further.

In practice, this study’s findings were used in the design of a community intervention, *Mtunze Mtoto Mchanga* (‘protect the newborn baby’), as part of the INSIST study for improved newborn care, which focuses on behaviour change communication through community volunteers. Overall, this paper contributes to a body of knowledge on thermal care practices, exploring the acceptability of these is rural southern Tanzania.
